# Antimicrobial efficacy of alternative compounds for use in oral care toward biofilms from caries‐associated bacteria in vitro

**DOI:** 10.1002/mbo3.695

**Published:** 2018-07-26

**Authors:** Fabian Cieplik, Esra Kara, Denise Muehler, Joachim Enax, Karl‐Anton Hiller, Tim Maisch, Wolfgang Buchalla

**Affiliations:** ^1^ Department of Conservative Dentistry and Periodontology University Medical Center Regensburg Regensburg Germany; ^2^ Oral Care Research Department Dr. Kurt Wolff GmbH & Co. KG Bielefeld Germany; ^3^ Department of Dermatology University Medical Center Regensburg Regensburg Germany

**Keywords:** antimicrobial, biofilm, cetylpyridinium chloride, chlorhexidine, citrus extract, dental caries

## Abstract

For caries‐active patients, antimicrobial measures may be useful in addition to mechanical biofilm removal. The aim of this study was to investigate the antimicrobial efficacy of alternative compounds for use in oral care from two main categories (i.e., preservatives and natural compounds) toward biofilms from caries‐associated bacteria as compared to oral care gold‐standards chlorhexidine digluconate (CHX), cetylpyridinium chloride (CPC), and zinc. Compounds were screened in initial *Streptococcus mutans* biofilms. Then, the most effective compounds were further investigated in mature *S. mutans* and polymicrobial biofilms comprising *Actinomyces naeslundii*,* Actinomyces odontolyticus,* and *S. mutans*. Here, distinct treatment periods and concentrations were evaluated. Biofilms were visualized by scanning electron microscopy and bacterial membrane damage was evaluated by means of flow cytometry and staining with SYBR Green and propidium iodide. Citrus extract was the only compound exhibiting similar antimicrobial efficacy in initial *S. mutans* biofilms (>5 log_10_) as compared to CHX and CPC, but its effect was clearly inferior in mature *S. mutans* and polymicrobial biofilms. Flow cytometric data suggested that the mechanism of antimicrobial action of citrus extract may be based on damage of bacterial membranes similar to CHX and CPC. From all alternative compounds investigated in this study, citrus extract exhibited the highest antimicrobial efficacy toward in vitro biofilms from caries‐associated bacteria, but still was less effective than oral care gold‐standard antiseptics CHX and CPC. Nevertheless, citrus extract may be a valuable antimicrobial compound for use in oral care for caries‐active patients.

## 1 INTRODUCTION

Dental caries affects about 2.5 billion adults and 573 million children worldwide and represents the most prevalent disease according to the 2015 Global Burden of Disease study (Kassebaum et al., 2017). Accordingly, untreated dental caries is a major economic burden for individuals and public health services (Kassebaum et al., 2015, 2017). It is well known that there is a great disparity with regard to distribution of dental caries, mostly by social standing and associated differences in diet, use of fluorides and social empowerment (Edelstein, 2006; Meyer et al., 2017; Oscarson, Espelid, & Jönsson, 2017; Selwitz, Ismail, & Pitts, 2007). In a recent cross‐sectional study in Norway, Oscarson et al. (2017) found that living in a rural area, low socioeconomic status, less frequent tooth cleaning and sugar containing soft drinks were associated with a higher prevalence of dental caries. Therefore, the development of more selective prevention concepts for these populations with strong caries incidence remains a major goal in contemporary preventive dentistry (Edelstein, 2006; Meyer et al., 2017; Oscarson et al., 2017). In these high‐risk patients, supportive antimicrobial measures may be useful in daily oral hygiene practice in addition to mechanical removal of biofilms and use of anticariogenic agents, for example, fluoride (ten Cate, 2009).

In this regard, several antiseptics such as chlorhexidine (CHX) or cetylpyridinium chloride (CPC) have been proposed, either applied as oral rinses, gels or varnishes, or incorporated into toothpastes (Haps, Slot, Berchier, & van der Weijden, 2008; Marsh, 2012; Walsh, Oliveira‐Neto, & Moore, 2015). However, the actual impact of such nonfluoride antimicrobials on caries prevention is still debated controversially (Walsh et al., 2015; Wang et al., 2017). For example, long‐term use of CHX, which can be regarded as gold‐standard antiseptic in oral care, exhibits some undesirable side effects like yellow‐brown staining of enamel surfaces and tongue and altering the sense of taste (Addy, Mahdavi, & Loyn, 1995; Autio‐Gold, 2008). These side effects may also result in poor compliance by patients in daily oral care (Cortellini et al., 2008; Fernandez y Mostajo et al., 2014). Furthermore, it has been recommended to limit the use of CHX to “those applications with a clear patient benefit” (i.e., intensive care) in order to reduce the risk of inducing acquired resistance toward CHX or even cross‐resistances toward antibiotics (Kampf, 2016). Accordingly, Kitagawa et al. (2016) recently reported development of enhanced tolerance toward CHX in *Enterococcus faecalis* as shown by increased minimum inhibitory concentrations (MICs) after 10 passages of treatment and regrowth. Likewise, Wang et al. (2017) found increased MICs in *E. faecalis*,* Streptococcus gordonii*,* Fusobacterium nucleatum,* and *Porphyromonas gingivalis* after 10 suchlike passages. Considering the wide use of CHX in dental practice and this risk of enhanced tolerance toward CHX in oral bacteria, it may be desirable to restrict its use and search for alternative antimicrobials for daily oral care with less strategic importance in general medicine (Kampf, 2016; Wang et al. (2017)).

Therefore, the aim of this study was to evaluate the antimicrobial efficacy of alternative compounds for use in oral care in vitro as compared to CHX, CPC, and Zinc, which all are antimicrobial substances frequently used in oral care and therefore served as positive controls here. Two different categories of compounds were tested, that is, (1) so‐called “gentle” and multifunctional preservatives and (2) natural products and essential oils. After screening in initial, 24 hr old *Streptococcus mutans* biofilms, the most effective compounds were then further evaluated in mature *S. mutans* biofilms and in polymicrobial biofilms cultured from *Actinomyces naeslundii*,* Actinomyces odontolyticus,* and *S. mutans*.

## 2 MATERIALS AND METHODS

### 2.1 Compounds

All compounds included in this study are listed in Table 1. All compounds were diluted in *aqua dest*. to reach the tested concentrations (all w/v). Measurements of pH were performed by a pH meter (HI 2211, Hanna Instruments, Vöhringen, Germany). In the case of citrus extract, pH adjustment was performed by adding Titripur^®^ sodium hydroxide solution (Merck KGaA, Darmstadt, Germany).

**Table 1:** Overview of compounds used in this study

Short name	Compound/INCI	Commercial name	Supplier	pH
Precare	Aqua, sodium levulinate, sodium benzoate	Cosphagard^®^ Precare	Cosphatec GmbH, Hamburg, Germany	6.1
Pretec	Phenoxyethanol, benzyl alcohol, phenylpropanol	Cosphagard^®^ Pretec		6.0
Presol	Phenoxyethanol, benzyl alcohol, phenethyl alcohol	Cosphagard^®^ Presol		9.8
PEA	Phenoxyethanol	Phenoxyethyl Alcohol	Symrise AG, Holzminden, Germany	7.3
SymDiol	1,2‐Hexanediol, 1,2‐Octanediol	SymDiol^®^ 68		5.3
SymSave	Hydroxyacetophenone	SymSave^®^ H		4.8
Zn^2+^	Zinc gluconate	Zinc gluconate	Sigma‐Aldrich, St. Louis, MO, USA	5.6
Oregano extract	*Origanum vulgare* extract	Extract of oregano leaves	Flavex Naturextrakte GmbH, Rehlingen‐Siersburg, Germany	5.3
Ajowan extract	*Trachyspermum ammi* extract	Extract of ajowan fruits		5.6
Clove bud extract	*Syzygium aromaticum* extract	Extract of clove flower bud		5.4
Myrrh extract	*Commiphora myrrha* extract	Extract of myrrh		5.0
Citrus extract	Glycerin, *citrus reticulata* fruit extract, *citrus aurantium amara* fruit extract, *citrus aurantium sinensis* peel extract, ascorbic acid, citric acid, lactic acid, water	BioSecur^®^ C320C	BIOSECUR LAB Inc., Mont St. Hilaire, Quebec, Canada	3.5, adjusted to 6.5
CHX	Chlorhexidine digluconate	—	Sigma‐Aldrich, St. Louis, MO, USA	7.9
CPC	Cetylpyridinium chloride	RonaCare^®^ CPC	Merck KGaA, Darmstadt, Germany	8.5

*Note*. INCI: international nomenclature of cosmetic ingredients.

### 2.2 Bacterial strains and culture conditions

*Streptococcus mutans* (DSM 20523), *A. naeslundii* (DSM 43013), and *A. odontolyticus* (DSM 19120) were obtained from DSMZ (Deutsche Sammlung von Mikroorganismen und Zellkulturen, Braunschweig, Germany). Bacteria were grown and maintained on Columbia Agar plates (provided by the Institute for Microbiology and Hygiene, University Medical Center Regensburg, Germany) under anaerobic conditions (80% N_2_, 10% CO_2_, 10% H_2_; microincubator MI23NK, SCHOLZEN Microbiology Systems, St. Margrethen, Switzerland). As a basal liquid medium, the modified Fluid Universal Medium (mFUM) was employed, supplemented with 67 mmol/L Sørensen's buffer (pH 7.2) and containing carbohydrate (0.15% (w/v) glucose and 0.15% (w/v) sucrose), as described earlier (Cieplik et al., 2018; Gmür & Guggenheim, 1983; Guggenheim, Giertsen, Schüpbach, & Shapiro, 2001). For the preparation of planktonic cultures, colonies were picked, suspended in 5 ml of mFUM with 0.5 ml fetal bovine serum (FBS; Gibco^®^ life technologies, Carlsbad, CA, USA) and cultured over‐night under anaerobic conditions in order to obtain bacteria in the stationary phase of growth. Afterward, cells were harvested by centrifugation (ROTINA 420 R, Hettich Lab Technology, Tuttlingen, Germany) and resuspended in mFUM yielding an optical density (OD) of 1.0, as measured by means of a spectrophotometer (Ultrospec 3300 pro, Amersham Biosciences, Amersham, UK). Bacterial suspensions were diluted 1:9 in the biofilm culture medium (BCM) consisting of 50% mFUM, 10% FBS and 40% whole unstimulated human saliva (saliva) that had been pooled from two volunteering authors (EK and DM; approved by the internal review board of the University of Regensburg, reference 17‐782_1‐101) and filter‐sterilized (pore size: 0.2 μm; Acrodisc^®^ Syringe Filters, Pall, Newquaw, UK).

### 2.3 Biofilm formation

Biofilms were formed in 96‐well polystyrene culture plates (Corning^®^ Costar^®^, Corning, NY, USA). For simulation of pellicle coating, wells were incubated with 200 μl saliva for 2 hr at room temperature. After that, saliva was discarded, and wells were filled with 200 μl of BCM containing *S. mutans* and incubated under anaerobic conditions. For initial *S. mutans* biofilms, biofilms were used for the antimicrobial assay after 24 hr. For mature *S. mutans* biofilms, medium was carefully exchanged after 24 and 48 hr and biofilms were used for the antimicrobial assay after a total culture period of 72 hr.

Polyspecies biofilms were cultured as it has been described in detail recently (Cieplik et al., 2018). Briefly, after discarding saliva, wells were filled with 200 μl of BCM containing *A*. *naeslundii* and *A*. *odontolyticus* and incubated under anaerobic conditions. After 16 hr, medium was carefully discarded and 200 μl fresh BCM containing *S. mutans* was added. After 48 hr, a further medium change was performed and biofilms were incubated for another 24 hr.

### 2.4 Antimicrobial assay

After the respective culture periods of 24 or 72 hr, medium was carefully discarded from the wells and the biofilms were either incubated with phosphate‐buffered saline (PBS; Biochrom, Berlin, Germany; untreated control) or with the respective test substances for 1, 3, or 10 min (50 μl each). Immediately afterward, PBS or the respective compound was carefully removed and each biofilm was brought to suspension with 200 μl of PBS and transferred to an Eppendorf tube. These were placed in an ultrasonic water‐bath chamber (35 kHz; Sonorex Super RK 102 H, Bandelin, Berlin, Germany) for 10 min and vortexed (REAX top, Heidolph Instruments, Schwabach, Germany) for 5 s to separate aggregated bacteria. Then, 10‐fold serial dilutions (10^−2^–10^−7^) were prepared in PBS.

Aliquots (3 × 20 μl for monospecies biofilms according to the method described by Miles et al. (Miles, Misra, & Irwin, 1938); 180 μl for polyspecies biofilms) were plated on Columbia blood agar and incubated anaerobically for 72 hr, whereupon colony‐forming units (CFU) were evaluated. For polyspecies biofilms, bacteria were differentiated visually by their respective colony morphology.

### 2.5 Scanning electron microscopy (SEM)

Polymicrobial biofilms were prepared on Permanox^®^ Chamber Slides (Nunc^®^ Lab‐Tek^®^ Permanox^®^, 4.2 cm^2^/well, Sigma‐Aldrich) as described above. After a culture period of 72 hr, biofilms were incubated with citrus extract 0.25%, CHX 0.2%, or CPC 0.1% for 10 min and the test substances were carefully discarded. Afterward, the samples were fixed by adding 2.5% glutaraldehyde buffered with Sørensen's phosphate buffer (0.1 M; pH 7.4) at room temperature for 2 hr. Then, the fixed samples were washed twice with PBS and three times with *aqua dest*. for 15 min each. Additional dehydration was performed using 30%, 50%, 70%, 80%, 90%, 96%, and 100% (v/v) graded ethanol, for 20 min each. After air‐drying overnight in a desiccator and removing the growth chamber, the slides were fixed on SEM stubs (Ø 25 mm). For coating, samples were purged with argon and sputtered with platinum for 30 s using a SCD 005 Sputter Coater (Bal‐Tec, Balzers, Liechtenstein). Biofilms were visualized in a SEM (Quanta 400 FEG; FEI Company, Hillsboro, OR, USA) in high vacuum mode at 2 kV with 6‐7 mm working distance. Tilt and focus were adjusted to ensure optimum viewing and images were taken from randomly selected fields on the slides.

### 2.6 Flow cytometric evaluation of membrane damage

For flow cytometry, SYBR green and propidium iodide (PI; both: Sigma‐Aldrich) were used as a fluorescent dyes to evaluate integrity of cytoplasmic membranes (Joux & Lebaron, 2000; Tawakoli, Al‐Ahmad, Hoth‐Hannig, Hannig, & Hannig, 2013). Mature (72 hr) *S. mutans* monospecies biofilms were prepared, treated, and resuspendized as described above. After that, samples were centrifuged once at 4300 *g* (MiniSpin, Eppendorf, Hamburg, Germany) for 3 min and resuspended in 1 ml PBS. Then, 10 μl of each sample was mixed with 984 μl PBS and 1 μl SYBR green (100×) and incubated for 15 min in the dark at room temperature. Subsequently, 5 μl PI (5 μg/ml) was added and incubated for another 5 min. Then, samples were immediately processed by a FACSCanto flow cytometer (Becton Dickinson, Franklin Lakes, NJ, USA) equipped with a 488 nm air‐cooled solid‐state laser with output of 20 mW. Green fluorescence emitted by SYBR green was detected on FL1, red fluorescence emitted by PI on FL3. Bacterial cells were gated on FSC/SSC dot plots from which FL1/FL3 dot plots were derived. In all cases, 10,000 events were evaluated.

### 2.7 Data analysis

CFU results are shown as medians, 1^st^, and 3rd quartiles and were calculated using SPSS for Windows, v. 25 (SPSS Inc., Chicago, IL, USA) from the values of at least six independent experiments, each performed in duplicate. In Figures, horizontal dotted and dashed lines represent reductions of 3 and 5 log_10_ steps of CFU, respectively, compared to the matching untreated control group UC. Medians on or below these lines demonstrate antimicrobial efficacy of at least 99.9% (3 log_10_) or 99.999% (5 log_10_), respectively, which is declared as biologically relevant antimicrobial activity or disinfectant effect, respectively, according to the guidelines of infection control (Boyce & Pittet, 2002).

Flow cytometric data were analyzed using FACSDiva™ software, version 5.0.2 (Becton Dickinson). The percentages of bacteria stained with SYBR green, PI, or both were calculated as medians, 1^st^, and 3rd quartiles from the values of six independent experiments by means of SPSS.

## 3 RESULTS

### 3.1 Screening for antimicrobial efficacy in initial (24 hr) *S. mutans* biofilms

**Figure 1:**
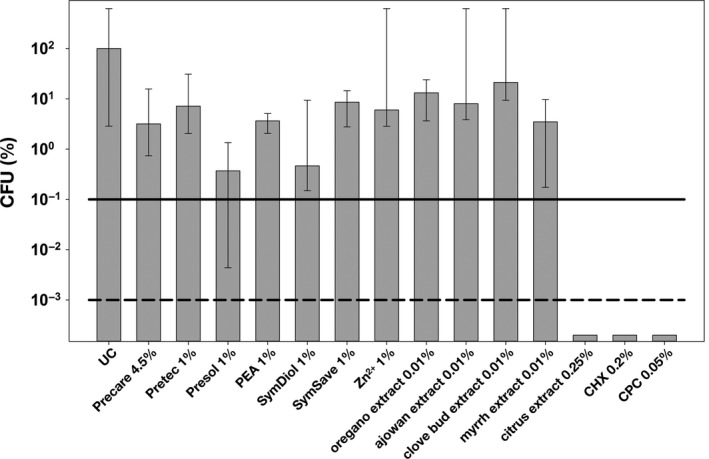
Screening of all compounds for their antimicrobial efficacy in initial (24 hr) *Streptococcus mutans* biofilms. All results are depicted as medians, 1st, and 3rd quartiles from six independent experiments, each performed in duplicate, on a log_10_‐scaled ordinate with untreated control group UC set to 100%. Horizontal dotted and dashed lines represent CFU‐reductions of 3 log_10_ and 5 log_10_, respectively. Incubation period was 10 min for all compounds

Figure 1 shows the results of the initial first screening round. Here, the antimicrobial efficacy of all compounds included in this study (Table 1) was investigated in initial (24 hr) *S. mutans* biofilms. Concentrations were chosen as the highest that were still recommended for use in oral care according to the respective manufacturers. The incubation period was set to 10 min.

Citrus extract 0.25%, CHX 0.2%, and CPC 0.05% were the only compounds that showed a pronounced antimicrobial efficacy reducing CFU by ≥5 log_10_ below the detection limit. The other eight compounds showed <3 log_10_ steps reduction in CFU: Presol 1% and SymDiol 1% showed CFU reductions of ≥2 log_10_, whereas all other compounds (i.e., Precare 4.5%, Pretec 1%, PEA 1%, SymSave 1%, Zinc 1%, Oregano extract 0.01%, Ajowan extract 0.01%, Clove bud extract 0.01%, Myrrh extract 0.01%) reduced CFU by <2 log_10_.

### 3.2 Antimicrobial efficacy of selected compounds in mature (72 hr) *S. mutans* biofilms

**Figure 2:**
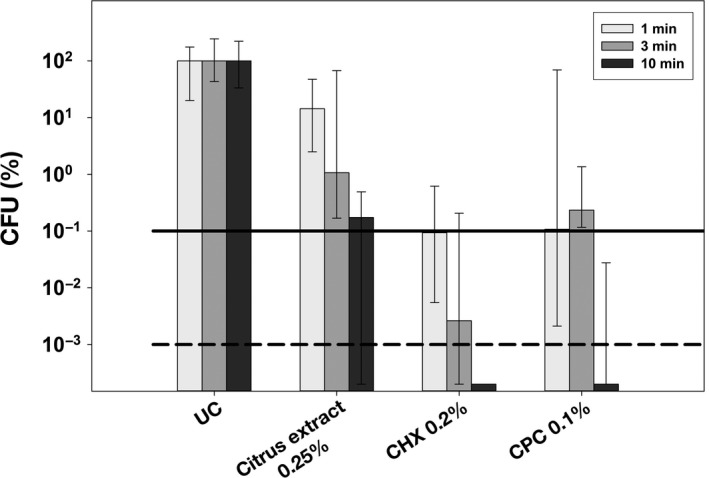
Antimicrobial efficacy of selected compounds citrus extract, CHX, and CPC in mature (72 hr) *S. mutans* biofilms. All results are depicted as medians, 1st, and 3rd quartiles from six independent experiments, each performed in duplicate, on a log_10_‐scaled ordinate with untreated control group UC set to 100%. Horizontal dotted and dashed lines represent CFU‐reductions of 3 log_10_ and 5 log_10_, respectively. Incubation periods were 1, 3, and 10 min for all selected compounds

The three most effective compounds citrus extract, CHX, and CPC were further investigated in mature (72 hr) *S. mutans* biofilms, applied for different treatment periods (1 min, 3 min, or 10 min) as shown in Figure 2. Untreated biofilms (UC) showed growth of 5 × 10^7^ to 2 × 10^8^ *S. mutans* cells. All compounds showed an effect of treatment period on antimicrobial efficacy. For citrus extract 0.25%, CFU‐reductions were 0.8 log_10_ after 1 min of incubation, 2 log_10_ after 3 min and 2.8 log_10_ after 10 min. Both, CHX 0.2% and CPC 0.1%, were more effective reducing CFU of *S. mutans* by 3 log_10_ after 1 min, by 4.6 log_10_ (CHX) or 2.6 log_10_ (CPC) after 3 min and by >5 log_10_ after 10 min.

### 3.3 Antimicrobial efficacy of selected compounds in polyspecies biofilms

**Figure 3:**
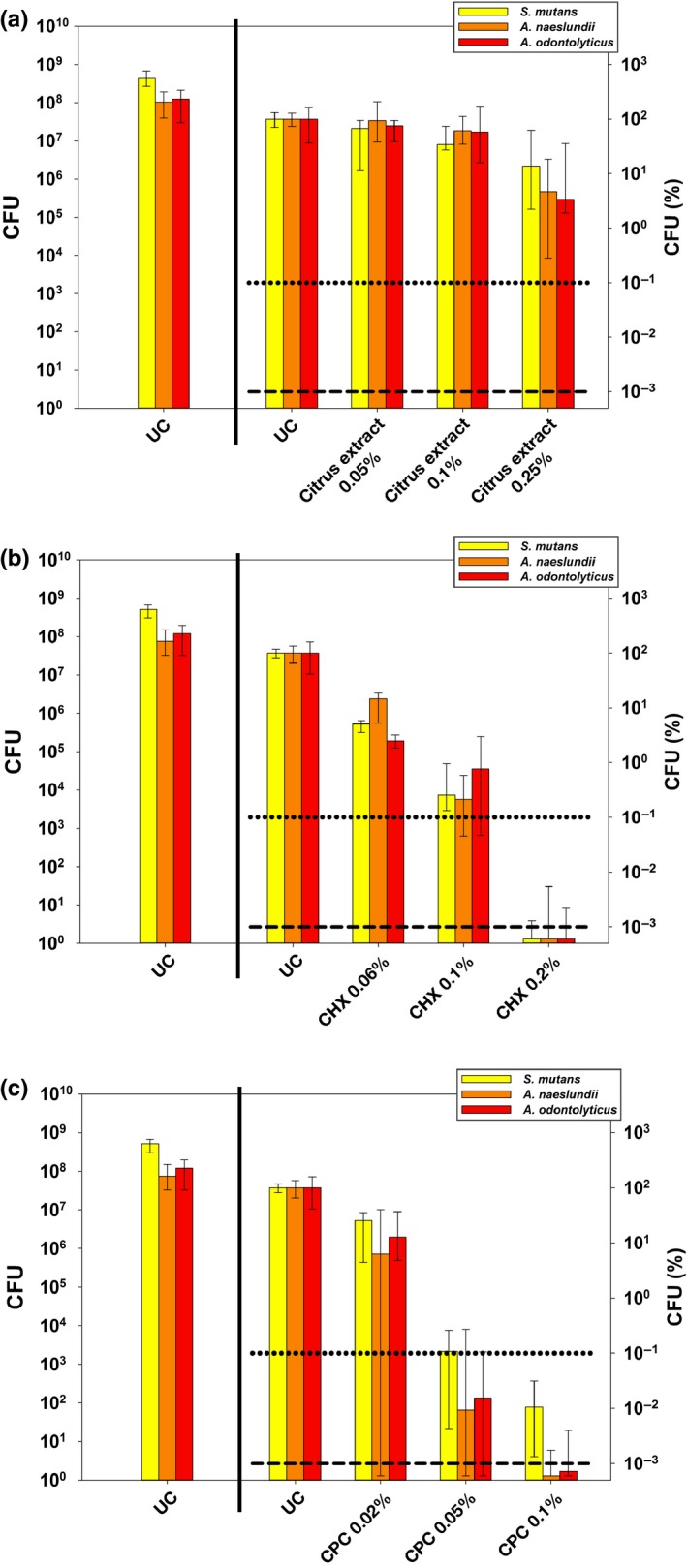
Antimicrobial efficacy of selected compounds citrus extract, CHX, and CPC in polyspecies biofilms from *Actinomyces naeslundii*,* Actinomyces odontolyticus,* and *S. mutans*. All results are depicted as medians, 1st, and 3rd quartiles from six independent experiments, each performed in duplicate, on a log_10_‐scaled ordinate. Left panels show untreated control groups UC as absolute CFU values. Right panels show relative CFU data (CFU [%]) with untreated control group UC set to 100% for each bacterial strain. Horizontal dotted and dashed lines represent CFU‐reductions of 3 log_10_ and 5 log_10_, respectively. (a) Antimicrobial efficacy of citrus extract in concentrations of 0.05%, 0.1%, and 0.25% after incubation for 10 min. (b) Antimicrobial efficacy of CHX in concentrations of 0.06%, 0.1%, and 0.2% after incubation for 10 min. (c) Antimicrobial efficacy of CPC in concentrations of 0.02%, 0.05%, and 0.1% after incubation for 10 min

The antimicrobial efficacy of citrus extract, CHX, and CPC was further assessed toward polyspecies biofilms comprising *S. mutans*,* A. naeslundii,* and *A. odontolyticus* applied in distinct concentrations for an incubation period of 10 min. Untreated polyspecies biofilms (UC) showed slightly more growth of *S. mutans* as compared to *A. naeslundii* and *A. odontolyticus*, as shown as absolute CFU values in the left panels of Figure 3. The right panels of Figure 3 depict relative CFU data (CFU (%)) with untreated controls (UC) set to 100% separately for each bacterial strain.

All compounds showed concentration‐dependent antimicrobial effects. Citrus extract (Figure 3a) exhibited ≤0.5 log_10_ reduction in CFU at 0.05% and 0.1%. At 0.25%, reductions were 0.9 log_10_ for *S. mutans*, 1.4 log_10_ for *A. naeslundii*, and 1.5 log_10_ for *A. odontolyticus*.

For CHX (Figure 3b) reductions in CFU were 0.8–1.6 log_10_ at 0.06% and 2.1–2.7 log_10_ at 0.1%. At 0.2%, CFU of all bacteria were reduced by > 6 log_10_ below the detection limit.

CPC (Figure 3c) revealed CFU reductions of 0.6–1.3 log_10_ at 0.02% and 3–4.2 log_10_ at 0.05%. At 0.1%, CPC reduced CFU of *S. mutans* by 4 log_10_, of *A. naeslundii* by 6 log_10_, and of *A. odontolyticus* by 5.2 log_10_.

### 3.4 Visualization of polyspecies biofilms by scanning electron microscopy

**Figure 4:**
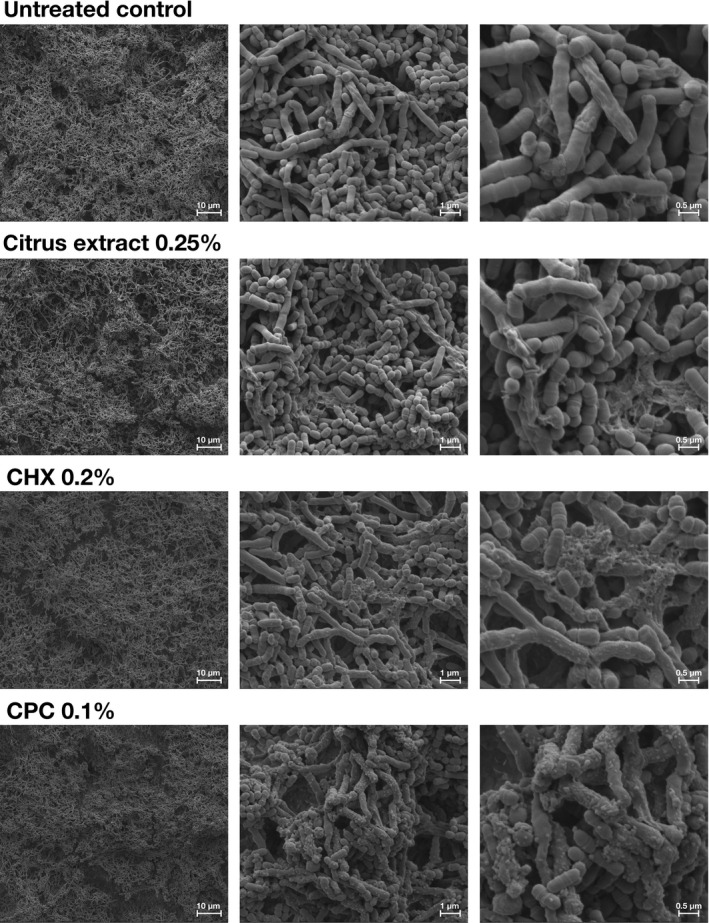
Exemplary visualization by means of scanning electron microscopy (SEM). Exemplary visualization of randomly selected fields of polyspecies biofilms (72 h), either untreated or treated with citrus extract 0.25%, CHX 0.2%, or CPC 0.1% in 3,000‐fold, 24,000‐fold, and 50,000‐fold magnification

Figure 4 shows exemplary SEM‐images taken from randomly selected fields of untreated polyspecies biofilms (untreated control) and biofilms treated with citrus extract 0.25%, CHX 0.2% or CPC 0.1%. Untreated biofilms showed a multilayered biofilm architecture. Treatments had no impact on overall biofilm structure. In biofilms treated with citrus extract 0.25%, cellular debris was visible, most likely resulting from dead bacterial cells. In samples treated with CHX 0.2% and in particular in samples treated with CPC 0.1%, vesicle‐like structures were apparent on the cell surfaces of bacteria.

### 3.5 Flow cytometric evaluation of membrane damage in mature (72 hr) *S. mutans* biofilms

**Figure 5:**
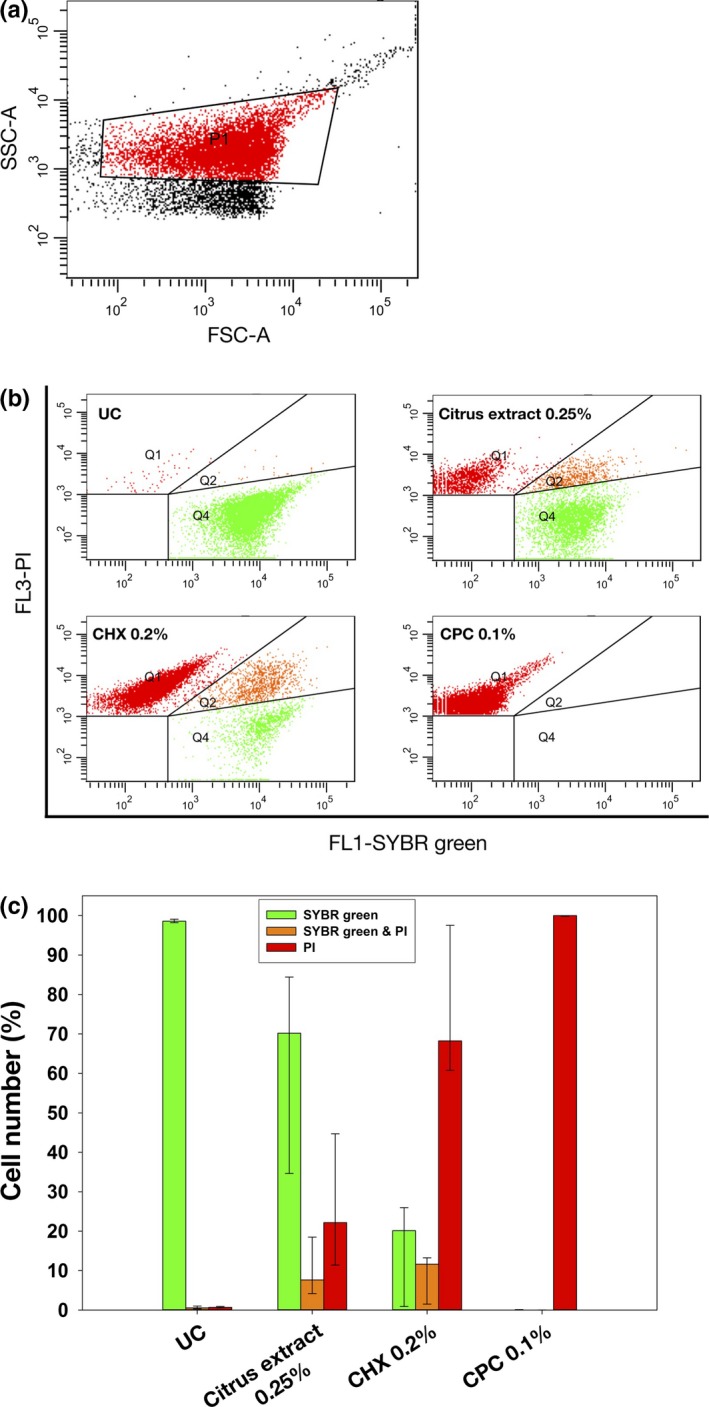
Flow cytometric evaluation of membrane damage in mature (72 h) *S. mutans* monospecies biofilms. (a) *S. mutans* cell population gated on dot plot FSC versus SSC with P1 showing the chosen region of interest. (b) Exemplary dot plots for untreated control (UC) and groups treated with citrus extract 0.25%, CHX 0.2%, or CPC 0.1%, respectively. Bacterial cells with intact membranes are shown in Q4 (stained with SYBR green only), whereas bacterial cells with damaged membranes (stained with PI) are shown in Q1. Q2 shows bacterial cells with damaged membranes but intact DNA (stained with SYBR green and PI) and therefore may represent damaged cells. (c) Summarized median percentages, 1st, and 3rd quartiles of bacterial cells stained with SYBR green (green) or PI (red) and double‐stained with SYBR green as well as PI are shown for untreated control (UC) and groups treated with citrus extract 0.25%, CHX 0.2%, or CPC 0.1%, respectively. Membrane damage could be observed after treatment with all compounds tested

Flow cytometry with SYBR green and PI as fluorescent dyes was employed to evaluate bacterial membrane damage in mature (72 hr) *S. mutans* biofilms. These dyes intercalate into DNA and exhibit a strong increase in fluorescence upon nucleic acid binding. While SYBR green stains all bacteria, PI is only able to stain bacteria with damaged membranes (Joux & Lebaron, 2000; Tawakoli et al., 2013). Figure 5a shows the chosen region of interest on dot plots FSC versus SSC, which was confirmed by SYBR green staining to discern between bacteria and cellular debris (data not shown). Figure 5b shows exemplary dot plots for all tested groups. Figure 5c shows summarized median percentages of bacterial cells stained with either SYBR green, PI or both for all groups. Untreated biofilms (UC) showed 99% bacteria stained with SYBR green. Biofilms treated with citrus extract 0.25% exhibited 22% bacteria stained with PI and 8% double‐stained bacteria, whereas biofilms treated with CHX showed 68% bacteria stained with PI and 12% double‐stained bacteria. In contrast, treatment with CPC 0.1% led to 100% PI‐positive bacteria.

## 4 DISCUSSION

The aim of this study was to investigate the antimicrobial efficacy of alternative compounds for use in oral care toward biofilms formed in vitro from caries‐associated bacteria. For this purpose, 11 compounds from two main categories, that is, (1) so‐called “gentle” and multifunctional preservatives and (2) natural compounds and essential oils, were evaluated and compared to contemporary gold‐standards frequently employed in oral care CHX, CPC, and Zn^2+^.

As a first step, all compounds were tested for their antimicrobial properties toward initial biofilms of *S. mutans* that had been cultured for 24 hr. *S. mutans* is known as key organism in the pathogenesis of dental caries due to its acidogenic and aciduric properties and even more due to its ability to produce high amounts of extracellular polysaccharides, which is known to be a virulence determinant of cariogenic biofilms (Klein, Hwang, Santos, Campanella, & Koo, 2015; Koo, Falsetta, & Klein, 2013). Here, all compounds were evaluated in commonly used concentrations recommended by their respective manufacturers for use in oral care. Although Zn^2+^ was included as a positive control known for its antibacterial action due to inhibition of bacterial adhesion and metabolic activity (Sheng, Nguyen, & Marquis, 2005), here it only exhibited small antimicrobial efficacy toward initial *S. mutans* biofilms (<2 log_10_). This is in line with recent data from the literature where it was concluded that very high concentrations of Zn^2+^ were needed to reach bactericidal effects, in particular toward biofilms (Gugala, Lemire, & Turner, 2017). However, besides this limited antimicrobial efficacy Zn^2+^ may still be a valuable compound for use in oral care products due to its proven efficacy in control of halitosis and its anticalculus effects (Sanz, Serrano, Iniesta, Santa Cruz, & Herrera, 2013).

So‐called “gentle” (i.e., nonirritating, paraben‐free) and multifunctional preservatives like the ones included in this study are usually added to cosmetic products in order to prevent bacterial growth and extend their shelf life, but mainly to offer additional benefits, for example, for skin soothing and moistening. Consequently, antimicrobial efficacy of such compounds is usually evaluated by testing minimum inhibitory concentrations (MICs) on planktonic bacteria or potential inhibitory effects on biofilm formation, whereas effects on already established biofilms are not in focus (Al‐Ahmad et al., 2008; Lalitha & Rao, 2013). Here, from all tested preservatives only two compounds (Presol 1%, SymDiol 1%) reduced CFU in *S. mutans* biofilms by ≥2 log_10_ steps, whereas all others were less effective (<2 log_10_).

Although there are many studies on antimicrobial efficacy of natural compounds and essential oils toward planktonic bacteria, studies on their efficacy toward biofilms are scarce (Freires, Denny, Benso, de Alencar, & Rosalen, 2015; Karygianni et al., 2015). Here, all four essential oils tested exhibited <2 log_10_ steps reduction in CFU only, when applied to initial *S. mutans* biofilms. Higher concentrations of the essential oils might have yielded superior antimicrobial efficacies but would be not applicable in oral care due to side effects (e.g., mucosal irritations or altering the sense of taste) and thus were not tested in this study.

From all compounds tested, only citrus extract 0.25% revealed a notable antimicrobial efficacy (>5 log_10_) toward initial *S. mutans* biofilms similar to the positive controls CHX 0.2% and CPC 0.05%. This compound is a commercially available product comprising extracts from various citrus fruits (i.e., *citrus reticulata*,* citrus aurantium amara,* and *citrus aurantium sinensis*), which has already been shown to have pronounced antimicrobial efficacy toward planktonic cultures of *Vibrio vulnificus* (Cormier, Scott, & Janes, 2013). In general, citrus fruits are known to be a rich source of phytochemicals, such as flavonoids and alkaloids, which show potent antimicrobial activity (Nogata et al., 2006; Oikeh, Omoregie, Oviasogie, & Oriakhi, 2016).

After this primary screening in initial *S. mutans* biofilms cultured for 24 hr, the most effective compounds citrus extract, CHX, and CPC were then further investigated for their antimicrobial efficacy toward mature *S. mutans* biofilms cultured for 72 hr. Here, all compounds were applied for clinically relevant treatment periods of 1 min, 3 min, or 10 min (resembling application of an oral mouthwash, brushing teeth or application of an oral healthcare gel, respectively) and their antimicrobial effects showed a clear effect of treatment period on antimicrobial efficacy. As compared to the positive controls CHX and CPC, citrus extract was less effective in these mature biofilms: While CHX 0.2% and CPC 0.05% still revealed >5 log_10_ steps reduction in CFU when applied for the longest treatment period of 10 min, citrus extract 0.25% showed a reduction by 2.8 log_10_ only.

The antimicrobial efficacy of CHX, CPC, and citrus extract was then further evaluated in a more complex polymicrobial biofilm model cultured from *A. naeslundii*,* A.  odontolyticus,* and *S. mutans*, whose culture conditions have been described and extensively discussed recently (Cieplik et al., 2018). While biofilms grown in vitro from defined consortia can never mimic the vast microbial complexity found in the oral cavity (Dewhirst et al., 2010; Marsh & Zaura, 2017), this may not be a crucial point for testing antimicrobial efficacy of given compounds because it is well‐known that the enhanced tolerance of bacteria in sessile biofilm mode is mostly due to the protective role of the extracellular polymeric substance (EPS), which acts as a diffusion barrier for antimicrobial agents (Bowen, Burne, Wu, & Koo, 2018; Stewart & Costerton, 2001). Here, all compounds were tested in three different concentrations and showed concentration‐dependent antimicrobial effects: At the highest respective concentrations tested, CHX 0.2% was the most effective compound (>5 log_10_ reduction in CFU of all species), followed by CPC 0.1% (>5 log_10_ of both *Actinomyces* spp., 4 log_10_ of *S. mutans*). Citrus extract 0.25% led to reductions of 0.9–1.5 log_10_ only. However, here it must be considered that citrus extract 0.25% was more effective than CHX and CPC at concentrations that are usually recommended for long‐term use in oral care, for example, in toothpastes (0.06% or 0.02%, respectively). Therefore, this compound may still be a valuable antimicrobial ingredient in oral care products.

The effects of citrus extract, CHX, and CPC on polyspecies biofilms were visualized by SEM. Here, treatment with citrus extract 0.25% resulted in some cellular debris on the top layer of the biofilms, whereas in addition vesicle‐like structures were apparent on bacterial cell surfaces after treatment with CHX 0.2% and even more after treatment with CPC 0.1% suggesting bacterial membrane damage. Kim et al. (2013) showed similar vesicle‐like structures after treatment of *E. faecalis* biofilms with CHX 2% for 5 or 10 min. Likewise, Shen et al. (2016) reported signs of cell lysis from their SEM examination of microcosm biofilms inoculated from subgingival plaque and treated with CHX 2% for 10 min. In contrast, SEM visualization of a polymicrobial biofilm comprising putative periodontal pathogens *A. naeslundii*,* Fusobacterium nucleatum,* and *Porphyromonas gingivalis* showed no suchlike vesicles, but cellular debris only after treatment with CHX 0.2% for 20 min (Cieplik et al., 2018). While Vitkov et al. (2005) found no alterations on the biofilm surfaces in SEM, they revealed from transmission electron microscopic investigations that treatment of ex vivo biofilms with CHX 0.1% for 5 min led to membrane blebbing associated with cytoplasmic vesicle‐like lesions, probably due to loss of cytoplasm through membrane perforations. Interestingly, here these vesicles were mostly found on *Actinomyces* cells, which may be due to their unique cell wall architecture, which makes them even resistant to treatment with lysozyme (Delisle, Barcak, & Guo, 2006). Therefore, this phenomenon may be strain dependent.

Based on these findings and for further investigation of bacterial membrane damage, flow cytometry was employed for analysis of mature *S. mutans* biofilms. The fluorescent stains SYBR green and PI were chosen, which both increase their fluorescence emission upon nucleic acid binding. While SYBR green enters all bacterial cells, PI is only able to enter cells with damaged membranes (Joux & Lebaron, 2000; Tawakoli et al., 2013). Both, CHX and CPC, revealed high percentages of PI‐positive or double‐stained bacterial cells, indicating membrane damage, which is in line with the SEM visualization. Furthermore, in the literature the mechanism of antimicrobial action of CHX and CPC is described as damage of bacterial membranes and subsequent leakage of cytoplasmic constituents (Cieplik et al., 2018; Jones, 1997; McDonnell & Russell, 1999). Citrus extract also led to an increase in PI‐positive cells as compared to the untreated control group and therefore may also target on bacterial membranes, as it is known from other essential oils and flavonoids (Paparella et al., 2008). The smaller increase as compared to CHX and CPC may be explained due to the smaller antimicrobial efficacy of citrus extract when applied for 10 min in these mature *S. mutans* biofilms (2.8 log_10_ vs. >5 log_10_).

## 5 CONCLUSIONS

In this study, several alternative antimicrobial compounds for use in oral care were evaluated for their antimicrobial efficacy toward biofilms formed from caries‐associated bacteria in vitro. As compared to the gold‐standards CHX and CPC, citrus extract was the only compound to be similarly effective in initial *S. mutans* biofilms (> 5 log_10_) but its antimicrobial effect was clearly inferior in mature *S. mutans* and polymicrobial biofilms. Nevertheless, citrus extract may still be a valuable natural alternative for use as an antimicrobial ingredient in oral care products. Thereby, its mechanism of antimicrobial action may be based on damage of bacterial membranes similar to CHX and CPC.

## CONFLICTS OF INTEREST

This study was funded in part by an industrial research grant from Dr. Kurt Wolff GmbH & Co. KG (Bielefeld, Germany) and one author (J. E.) is an employee of this company. The authors declare that there are no other conflicts of interest.

## DATA ACCESSIBILITY STATEMENT

The data presented in this manuscript are available at request from the corresponding author's institution archives.
